# Correlates of HIV Stigma in prison guards and case workers in a national prison system

**DOI:** 10.1186/1742-4690-9-S1-P67

**Published:** 2012-05-25

**Authors:** Carmen E Albizu-García, Cynthia Pérez-Cardona, Salvador Santiago-Negrón

**Affiliations:** 1 School Public Health University Puerto Rico, San Juan, Puerto Rico

## 

HIV is over-represented in correction facilities. Health organizations have provided a framework for effective response to HIV/AIDS in prisons yet many countries lack prison-based HIV prevention and treatment. Prison workforce’s fears of HIV contagion and stigma have been identified as barriers to appropriate responses. We explore the magnitude of stigma towards HIV in correctional officers and case workers in prison system across workforce categories, sex, and HIV knowledge.

A self-administered, anonymous questionnaire was completed by 95 case workers and 214 correctional officers from 26 of the 39 penal institutions in Puerto Rico (PR) selected multi stage random sample design. Stigma towards HIV was assessed with a Spanish measure of stigma towards HIV among health professionals containing 18 items measured with 6 categories agreement scale. Predictor variables include demographics, work experience in corrections, and HIV knowledge. The study was approved by the IRB. We report the scale’s reliability and the weighted mean and standard deviation of responses to the stigma measure for the total sample and by workforce category, sex, and HIV knowledge.

Of the 309 respondents 58% were male and 68% report 5 or more years iof employment. Scale reliability as measured with Cronbachs alpha was 0 .81. The values of the stigma towards HIV measure ranged from 18-108, with higher scores corresponding to greater stigma. The mean of the stigma score for the total sample was 69.5 (SD 15.6). The means did not differ significantly when compared for correctional officers vs. case workers or by sex, yet an inverse relationship was found between stigma and HIV knowledge.

Stigma scores fell within the second highest quartile suggesting that HIV stigma is high in this sample. The availability of a culturally sensitive instrument in Spanish contributes to work in the field and the findings underscore the need to address stigma towards HIV in the prison workforce in this and other contexts. Research should be conducted on the effect of HIV knowledge gains on stigma reduction in this population.

